# Prevalence of coccidiosis among village and exotic breed of chickens in Maiduguri, Nigeria

**DOI:** 10.14202/vetworld.2016.653-659

**Published:** 2016-06-25

**Authors:** Jallailudeen Rabana Lawal, Saleh Mohammed Jajere, Umar Isa Ibrahim, Yaqub Ahmed Geidam, Isa Adamu Gulani, Gambo Musa, Benjamin U. Ibekwe

**Affiliations:** 1 Department of Veterinary Medicine, Faculty of Veterinary Medicine, University of Maiduguri, PMB 1069, Maiduguri, Borno State, Nigeria; 2 Department of Veterinary Public Health and Preventive Medicine, Faculty of Veterinary Medicine, University of Maiduguri, PMB 1069, Maiduguri, Borno State, Nigeria

**Keywords:** coccidiosis, exotic breeds, Maiduguri, Northeastern Nigeria, prevalence, village chickens

## Abstract

**Aim:**

Coccidiosis is an important enteric parasitic disease of poultry associated with significant economic losses to poultry farmers worldwide. This survey was conducted from June 2014 through July 2015 with the main goal of investigating the prevalence and associated risk factors of coccidiosis among village and exotic breeds of chickens in Maiduguri, Northeastern Nigeria.

**Materials and Methods:**

A total of 600 fecal samples from live and slaughtered birds comprising 284 young, 141, growers and 175 adult birds; 379 male and 221 female birds; 450 exotic and 150 local breeds of birds were randomly collected either as bird’s fresh droppings or cutting open an eviscerated intestine of slaughtered birds, while noting their age, sex, and breeds. Samples were analyzed using standard parasitological methods and techniques.

**Results:**

An overall prevalence rate of 31.8% (95% confidence interval: 28.07-35.52) was obtained. Higher prevalence rates were recorded in growing birds 58.9% (50.78-67.02), female birds 35.3% (29.00-41.60), exotic birds 42.4% (37.83-46.97), and broiler birds 68.7% (61.28-76.12). Similarly, higher infection rates were also observed among birds sampled from Mairi ward 66.7% (56.03-77.37), intensive management system 46.5% (41.61-51.39), and constructed local cages 54.0% (46.02-61.98). The difference in prevalence of coccidiosis among age groups, breeds, among exotic breeds, sampling sites, husbandry management systems, and litter management systems was statistically significant (<0.0001). However, no significant difference (p>0.05) of infection rates was observed in sex.

**Conclusion:**

Coccidiosis is endemic in both commercial and backyard poultry farms in Maiduguri due to poor management practices encouraging *Eimeria* oocysts build-up. It is therefore, recommended that poultry farmers should practice strict biosecurity measures on their farms, creating awareness on the prevalence of coccidiosis, routine vaccination against coccidiosis and educating poultry farmers on the need for maintaining good hygienic standards and good flock health management.

## Introduction

In Nigeria like in most developing nations, chickens are the most important class of the poultry species in terms of number and rate of investment in poultry production [1]. The exotic breeds are usually managed intensively either in battery cages or deep litter system of management, while the village chickens are reared extensively; where they are allowed to scavenge food for survival. Poultry’s meat and eggs continue to be the major sources of protein for the rapidly expanding population worldwide. This is due to low production costs as compared with livestock farming and absence of religious restrictions on the poultry meat in both developing and developed nations. Poultry coccidiosis has been reported as a major constraint to successful commercial and backyard poultry farming due to its significant high mortality rates and huge economic losses globally.

Poultry coccidiosis, caused by the protozoan parasite of the genus *Eimeria*, remains one of the most important parasitic diseases in poultry industry worldwide [2-4]. Several studies established the prevalence and economic importance of coccidiosis as a major parasitic disease in both local and exotic breeds of poultry worldwide [5-7]. The prevalence of coccidiosis was reported in many countries such as Iran [2], Egypt [8], Ethiopia [9], India [10], South Africa [11], and Nigeria [7,12,13].

About 1800 *Eimeria* colonize and infect the intestinal tract of different animals and birds [14] and infection with this parasite normally occurs through ingestion of feed or water contaminated with sporulated oocysts [15]. About nine species of *Eimeria* have been recognized in domesticated chickens, of which *Eimeria brunette*, *Eimeria maxima*, *Eimeria necatrix*, *Eimeria tenella* are the most pathogenic; *Eimeria acervulina*, *Eimeria mitis*, *Eimeria mivati* are the less pathogenic and *Eimeria praecox* and *Eimeria hagani* are the lesser pathogenic [4,16]. Coccidiosis resulting from the pathogenic *Eimeria* species is usually characterized by dysentery, enteritis, diarrhea, which may be bloody with certain *Eimeria* species, emaciation, lower feed conversion rate, delayed sexual maturity, drooping wings, poor growth and low production [17,18] with attendant high mortality and morbidity rates [19]. The most common and pathogenic species that affects the poultry industry globally is the *E. tenella* [20], with 100% morbidity and a high mortality due to extensive damage of the digestive tracts of chickens [6]. Mortality rates are usually high in young chicks, because most of the *Eimeria* species affects birds between the age of 3 and 18 weeks [2,7].

The occurrence of different *Eimeria* species combinations and the intensity of infection vary considerably, both locally and globally [14,21]. High incidence of coccidiosis is usually observed in poultry managed under intensive management system like deep litter due to increased likelihood of high oocysts accumulation in the litters [7,22]. Furthermore, higher stocking densities have been linked with increased incidence of coccidiosis due to a higher rate of infection and transmission of the coccidian oocysts in dense flocks from one poultry house to another [23]. Indiscriminate use of anticoccidial drugs in feed and water has led to serious drug resistance problems [24].

The traditional control of coccidiosis mainly relies on chemoprophylaxis, which appeared to be effective in the last decades. However, the increased occurrence of resistance against routine anti-coccidial drugs has left the poultry industry with a renewed challenge for coccidiosis prevention and control and propelled the search for alternative strategies among which vaccination is of major importance [25,26]. There are currently few studies on the prevalence and risk factors associated with the occurrence of poultry coccidiosis in Maiduguri, despite the fact that majority of the populace practice backyard poultry farming for petty cash. More so, the increasing demand of chicken as a source of protein (meat and egg) by the increasing human population saw an unprecedented growth and expansion of the poultry industry in Maiduguri. Therefore, this study was conducted to determine the prevalence of coccidiosis among exotic and village chickens as well as identify the risk factors associated with its occurrence in Maiduguri.

## Materials and Methods

### Ethical approval

Ethical approval for the present study was duly obtained from and approved by the Institutional Animal ethics and Research committee of the Faculty of Veterinary Medicine, University of Maiduguri, Maiduguri, Borno State, Nigeria.

### Study area

Maiduguri is the capital and largest city of Borno State, located within the Sahel savannah zone of the Northeastern Nigeria. It lies approximately between 11° 5’ and 11.83° N latitude and 13° 09’ and 13.50° E longitude at about 350 m (1161 ft) above sea level with ambient temperatures of 40-45°C (http://www.unimaid.edu.ng/About_Maid.aspx). The climate is hot and dry for a greater part of the year with a rainy season from June to September in the Northern part and May to October in the Southern part with a mean annual rainfall and temperature of about 650 mm and 32°C, respectively. The mean relative humidity ranges from 30% to 50% with the minimum usually experienced in the months of February and March, when it drops to as low as 10% and reaches maximum in August, as high as 90%.

### Husbandry details for the different poultry management systems

#### Intensive system

This system is not usually practiced by peasant poultry farmers due to its expensive nature. It comprises battery cage, deep litter system, or locally constructed cage system. Here, birds are housed throughout their life cycle in cages with the provision of good feed concentrates, litters of sawdust, feederers and waterers for their feeding and watering. Good attention is given on their vaccination schedule throughout their lives, good disease control and prevention programs, etc. However, because of the possibility of water spillage onto the litters and subsequently moisture and humidity development makes this system at high risk of coccidiosis. Coccidiosis is commonly seen in this system compared with others.

#### Semi intensive system

Most of the chickens reared under this system scavenge for most of their feeds and other nutritional feed sources outside with provision of shelter. Normally kept housed in the night to roost and released the following morning to scavenge. There is some form of attention with regard to the provision of feed supplements, vaccination and other disease preventive measures and provision of shelter. The housing could vary from locally constructed wirehouse to spare room or store in the house. Mortality from diseases and lost from theft are usually low under this system of production.

#### Extensive system

This is mainly seen in African countries were the majority of the rural households practice it. In Nigeria, most of the rural households practice this system of production. It is characterized with family ownership of birds. Here, the birds are left to scavenge outside for food and other nutritional needs. The housing may or may not be provided. Low productivity, poor feeding and disease control characterized this system. Birds are left with no vaccinations at any stage of their lives and exposed to varying levels of diseases. Under this system, there is no attention to feeding birds with good feeds; disease control and good shelter. There is high mortality from diseases, predators, and theft.

### Sample collection

Simple random sampling method was used to collect fecal samples from poultry dressing slabs in Maiduguri Monday Market, Tashan Bama market and custom markets as well as from the Government Reserve Area (GRA), Mairi ward, University of Maiduguri (UM) research poultry farms, backyard poultry houses in and around UM senior and junior staff quarters. Pooled fecal samples were aseptically collected from bird’s fresh droppings in poultry farms or cutting open freshly eviscerated intestine of slaughtered chickens, squeezing out the feces into a sterile labeled polythene bags and immediately transported to the Veterinary Medicine Laboratory, Department of Veterinary Medicine, Faculty of Veterinary Medicine for further parasitological analysis. Age, sex and breed of chickens, from which sample was collected, were recorded at the time of sampling.

### Microscopic examination of gut samples

All the intestines and ceca were examined carefully for the presence of external lesions. The intestines were cut opened using sterile scalpel blade, and the gut contents were microscopically examined by direct wet mount smear method for the presence of *Eimeria* oocysts as described by Soulsby [27]. The results for the presence or absence of *Eimeria* oocysts were recorded. When no oocyst is found on the three slides of a single sample, it is recorded as a negative sample. The positive samples were separated and kept in a 2.5% aqueous solution of potassium dichromate (K_2_Cr_2_O_7_) for sporulation as described by Al-Quraishy *et al*. [28].

### Microscopic examination of fecal samples

The fecal samples were soaked overnight at 37°C in 2.5% (w/v) aqueous solution of potassium dichromate. The samples were shaken vigorously to break up the feces. The suspension was filtered through cheesecloth into a beaker. The filtrate obtained was centrifuged at 447 ×*g* (Rotor radius = 10) for 5 min to settle down the oocysts. The supernatant fluid was discarded and the *Eimeria* oocysts present in the sediment were separated using floatation technique and then examined carefully through microscope using oil emersion lens for the presence of the *Eimeria* oocysts. Counting of oocysts was done using McMaster counting technique and was expressed as per gram of feces [29].

### Statistical analysis

Data were collected and analyzed initially in Microsoft office Excel version 2011 to obtain percentages and prevalence of coccidian oocysts. The prevalence (P) in percentage was calculated using the formula P = d/n, where d is the number of positive samples analyzed at that point in time and n is the total number of chickens sampled at that point in time [30]. The SPSS statistical software version 22 was used for both Fisher’s exact test and Chi-square statistical analysis. The statistically significant association between the risk factors and the infection was determined at p<0.05.

## Results

Of the 600 samples tested, 191 were positive, given an overall prevalence rate of 31.8% (95% confidence interval: 28.07-35.52) (Table-1). Age-specific prevalence rate revealed a statistically significant difference (<0.0001) across the age groups studied. The high prevalence rate of 58.9% (50.78-67.02) was observed among growers as compared with respective prevalence of 36.3% (30.71-41.89) and 2.9% (0.41-5.39) among young and adult birds (Table-1). Female birds had the high prevalence of coccidiosis 35.3% (29.00-41.60) as compared with male birds 29.8% (25.20-34.40). However, this was not statistically significant at p>0.05 (Table-1). Based on breeds, exotic birds had high prevalence of 42.4% (37.83-46.97), and none was observed among the local/village breeds (Table-1). Within breed prevalence reveals high prevalence of 68.7% (61.28-76.12) among the broiler breeds (<0.0001). While respective prevalence rates of 55.3% (47.34-63.26) and 3.3% (0.44-6.16) were observed among the pullets and layer breeds (Table-1).

**Table-1:** Risk factors associated with avian coccidiosis in Maiduguri, Northeastern Nigeria (n=600).

Risk factors	Number of examined	Number of positive	Prevalence (95% CI)	χ^2^	p value
Age (weeks)					
Young (1-4)	284	103	36.3 (30.71-41.89)	117.8	<0.0001
Grower (5-16)	141	83	58.9 (50.78-67.02)		
Adult (>16)	175	5	2.9 (0.41-5.39)		
Sex					
Male	379	113	29.8 (25.20-34.40)	1.9	0.165
Female	221	78	35.3 (29.00-41.60)		
Breed					
Exotic	450	191	42.4 (37.83-46.97)	93.4	<0.0001
Local/village	150	0			
Exotic species*					
Broiler	150	103	68.7 (61.28-76.12)	146.3	<0.0001
Pullet	150	83	55.3 (47.34-63.26)		
Layer	150	5	3.3 (0.44-6.16)		
Overall	600	191	31.8 (28.07-35.52)		

* Total examined was 450. CI=Confidence interval

According to the sampling sites, the high prevalence rate of coccidiosis 66.7% (56.03-77.37) was observed in Mairi ward (<0.0001). While prevalence of 59.7% (50.58-67.82), 42.4% (33.74-51.06) and 18.7% (9.88-27.52) were observed from UM junior staff quarters, UM senior staff quarters and GRA, respectively (Table-2). However, none was observed from UM Poultry Research Farms and Poultry dressing slabs (Table-2).

**Table-2:** Prevalence of avian coccidiosis according to various sampling sites in Maiduguri metropolis, Northeastern Nigeria.

Sampling sites	Number of examined	Number of positive	Prevalence (95% CI)	χ^2^	p value
Mairi	75	50	66.7 (56.03-77.37)	86.6	<0.0001
UM junior staff quarters	125	74	59.2 (50.58-67.82)		
UM senior staff quarters	125	53	42.4 (33.74-51.06)		
GRA	75	14	18.7 (9.88-27.52)		
UM poultry research farm	50	0	-		
Poultry dressing slab	150	0	-		
Overall	600	191	31.8 (28.07-35.52)		

UM=University of Maiduguri, GRA=Government Reserve Area, CI=Confidence interval

Based on the husbandry system, the high prevalence of coccidiosis 46.5% (41.61-51.39) was observed in the intensive system as compared with 10.0% (1.68-18.32) in the semi-intensive system (p<0.05). However, none was observed in the extensive management system (Table-3). A statistically significant prevalence rate of 54.0% (46.02-61.98) was observed in the constructed local cages as compared with 27.5% (23.12-31.88) in the deep litter systems. However, none was observed in samples collected from birds raised under the battery cage system (Table-4).

**Table-3:** Prevalence of coccidiosis according to husbandry systems in Maiduguri metropolis, Northeastern Nigeria.

Husbandry system	Number of examined	Number of positive	Prevalence (95% CI)	χ^2^	p value
Intensive	400	186	46.5 (41.61-51.39)	116.6	<0.0001
Semi-intensive	50	5	10.0 (1.68-18.32)		
Extensive	150	0	-		
Overall	600	191	31.8 (28.07-35.52)		

CI=Confidence interval

**Table-4:** Prevalence of coccidiosis according to litter systems in Maiduguri metropolis, Northeastern Nigeria.

Litter system	Number of examined	Number of positive	Prevalence (95% CI)	χ^2^	p value
Constructed local cage	150	81	54.0 (46.02-61.98)	60.8	<0.0001
Deep litter	400	110	27.5 (23.12-31.88)		
Battery cage	50	0	-		
Overall	600	191	31.8 (28.07-35.52)		

CI=Confidence interval

## Discussion

Poultry continues to be the major source of cheap protein in the form of chicken meat and eggs as well as a source of petty cash in developing countries. Coccidiosis is the most common enteric parasitic disease of poultry and a major constraint to successful poultry farming worldwide. The coccidian parasite, *Eimeria* has special preferences to the chicken’s intestinal tract with predilection in different anatomical sites of the tract associated with bloody diarrhea, low productivity due to low feed conversion rates (production losses), reduced welfare of birds and increased mortality resulting from the extensive damage of the gastrointestinal tracts. This specificity vary with the species of *Eimeria* present. The overall prevalence of 31.8% was obtained in this study. This is lower than 36.6% prevalence rate as reported by Dakpogan and Salifou [7] in Benin, 37.1% reported by Jatau *et al*. [13] in Zaria, and 69% by Olanrewaju and Agbor [31] in Abuja. However, it is higher than 11.4% reported by Grema *et al*. [32] in Gombe and 14% by Adamu *et al*. [33] in Sokoto state. It is similar to 31% observed by Lunden *et al*. [23]. The variation in the reported prevalence might be attributed to different factors such as sampling periods, sample size, geographical area, and climatic conditions observed in the different study areas. It should be borne in mind however, that incidence of coccidiosis is high in highly humid geographical areas explaining the higher prevalences reported in different parts of Nigeria. The relatively high prevalence reported in this work could also be explained by the poor management practices in both the commercial and backyard poultry farming. One of these practices as observed by the researchers during sampling is the perpetual spillage of water on the litters from the poultry drinkers, which encourages *Eimeria* build-up and subsequent sporulation and infection. This is consistent with the reports by Methusela *et al*. [34] and Taylor *et al*. [35].

The prevalence rate of coccidiosis was high in growers and younger birds compared with adult birds (Table-1). This agrees with the study by Omer *et al*. [36] who reported that all ages of birds are susceptible to coccidiosis, but younger birds are more susceptible to infection than older birds. However, it is parallel with studies by Amare *et al*. [37], Etuk *et al*. [38], and Dakpogan and Salifou [7], where both reported high prevalence rates in adult birds than in other age brackets. This might be associated with the immature immune system in young birds leaving them susceptible to infection even with the lower or less pathogenic strain of *Eimeria* species. In addition, since chicks are not immunized against coccidiosis, they can experience higher mortality rates in an *Eimeria* outbreak as observed by Chapman *et al*. [39]. Similarly, young chicks and growing birds in commercial poultry farms are at greater risk of coccidiosis outbreak, because they are usually kept under deep litters made of wood shavings for several weeks during the brooding period. Moreover, in a poorly managed poultry farm settings where different age groups of birds are stocked in the same deep litter pen as observed in this study, all the age groups of birds remains at high risk of infection with coccidia.

Exotic breeds had high prevalence rate (Table-1). In contrast, none was observed from the scavenging village chickens and those caged ready for slaughter in the poultry markets (Table-1). This agrees with studies by Gari *et al*. [40] and Oljira *et al*. [9]. Breed factors in the exotic breeds compared with the local/village chickens could explain the high infection rates observed. The scavenging village chickens are also less likely to ingest pathogenic level of the coccidian oocysts during feeding. The infection rate was relatively higher in female birds as compared with males (35.3% vs. 29.8%). However, the rates are not statistically different (Table-1) indicating both sexes have equal chances of acquiring and becoming infected with *Eimeria* oocysts during feeding or in an outbreak scenario, which is consistent with previous studies [9,40,41].

Broiler breeds had high coccidian infection rate (68.7%) compared with pullets (55.3%) and layers (3.3%) (Table-1). This might be connected with higher stocking densities and intensive husbandry management systems practiced in broiler production in the study area. This is in line with reports by Nnadi and George [22] in Southeastern Nigeria; Nematollahi *et al*. [2] in Iran; Jatau *et al*. [13] in Zaria, Northwestern Nigeria and Naphade [42] in India. However, others reported high prevalence among laying birds [7,43].

In this study, we reported no coccidian infection in birds sampled from UM research poultry farms - where birds were kept in battery cages and the village chickens sampled from the various poultry dressing slabs (Table-2). However, most of the fecal samples positive for coccidian oocysts were sampled from intensively managed poultry farms with deep litter systems and constructed local cages in the UM senior staff/junior staff quarters, GRA and Mairi ward (Tables-3 and 4). This finding is not surprising, since most of the poultry farms do not maintain strict biosecurity measures and good management practices such as maintaining standard hygienic and sanitary measures. Good management practices in poultry farms reduce the risk for *Eimeria* oocysts build-up and sporulation resulting from spillage of water and humid environment. This agrees with previous reports indicating high coccidian burden among birds raised under intensive management systems particularly in the deep litter management, resulting from relatively high oocysts accumulation within the deep litters [7,34,35].

## Conclusion

The prevalence of coccidiosis was found low in poultry farms that practice high standard of hygiene in the study area. However, high burden of coccidian infection was recorded in farms that were careless in observing adequate hygienic measures. Poor management practice is the main risk factor favoring the onset of coccidiosis such as oocysts build-up, oocysts sporulation, and the humid environment. It is, therefore, recommended that strict biosecurity measures such as changing of clothes by attendants from one farm to another or attending different poultry houses within same farms to avoid transmission of oocysts to naive poultry pens or farms; avoiding water spillage in the poultry pens, wild birds should be kept far away from and around the poultry houses; use of anticoccidial drugs as an effective control measure against coccidiosis; Poultry farmers are strictly advised to consult registered veterinarians routinely for professional advice; good management practices are the handy tools to minimize the occurrence and spread of coccidiosis; maximum hygienic measures should be practiced in handling utensils (like feeding and watering troughs) used in poultry houses; routine vaccination schedule for coccidiosis should be introduced, and if available, attenuated or live vaccines should be used for effective prevention and control of coccidiosis in the study area.

## Authors’ Contributions

Authors UII and YAG conceived and designed the study. JRL and SMJ conducted the study. IAG and GM contributed in literature review and some aspect of laboratory work. BUI did the sampling. JRL and SMJ did the data analysis and drafted the manuscript. UII and YAG revised the manuscript. All authors read and approved the final manuscript.
